# Significant blockade of multiple receptor tyrosine kinases by MGCD516 (Sitravatinib), a novel small molecule inhibitor, shows potent anti-tumor activity in preclinical models of sarcoma

**DOI:** 10.18632/oncotarget.6547

**Published:** 2015-12-10

**Authors:** Parag P. Patwardhan, Kathryn S. Ivy, Elgilda Musi, Elisa de Stanchina, Gary K. Schwartz

**Affiliations:** ^1^ Herbert Irving Comprehensive Cancer Center, Columbia University, New York, NY, USA; ^2^ Department of Molecular Pharmacology and Chemistry, Memorial Sloan Kettering Cancer Center, New York, NY, USA; ^3^ Department of Medicine, Division of Hematology/Oncology, Columbia University Medical Center, New York, NY, USA

**Keywords:** MGCD516, Sitravatinib, tyrosine kinase, imatinib, crizotinib

## Abstract

Sarcomas are rare but highly aggressive mesenchymal tumors with a median survival of 10–18 months for metastatic disease. Mutation and/or overexpression of many receptor tyrosine kinases (RTKs) including c-Met, PDGFR, c-Kit and IGF1-R drive defective signaling pathways in sarcomas. MGCD516 (Sitravatinib) is a novel small molecule inhibitor targeting multiple RTKs involved in driving sarcoma cell growth. In the present study, we evaluated the efficacy of MGCD516 both *in vitro* and in mouse xenograft models *in vivo*. MGCD516 treatment resulted in significant blockade of phosphorylation of potential driver RTKs and induced potent anti-proliferative effects *in vitro*. Furthermore, MGCD516 treatment of tumor xenografts *in vivo* resulted in significant suppression of tumor growth. Efficacy of MGCD516 was superior to imatinib and crizotinib, two other well-studied multi-kinase inhibitors with overlapping target specificities, both *in vitro* and *in vivo*. This is the first report describing MGCD516 as a potent multi-kinase inhibitor in different models of sarcoma, superior to imatinib and crizotinib. Results from this study showing blockade of multiple driver signaling pathways provides a rationale for further clinical development of MGCD516 for the treatment of patients with soft-tissue sarcoma.

## INTRODUCTION

Sarcomas are a heterogeneous group of rare tumors arising from embryonic mesodermal origin and presenting as soft tissue and bone tumors [1, 2]. Despite making up only about 1% of all cancer cases each year (about 14,000 cases), there are more than 50 currently characterized types [1, 3–5]. The most common sarcomas include leiomyosarcoma (20%), liposarcoma (17%) and synovial sarcoma (14%) [6]. Other rare sarcomas, such as malignant peripheral nerve sheath tumor, have an incidence rate of about 5% [7]. First line of treatment for localized disease is surgical resection followed by radiotherapy with or without adjuvant chemotherapy. Metastatic disease, however, requires intensive chemotherapy with great toxicity and generally inadequate efficacy, leading to a median survival from diagnosis of 10–18 months [8].

Recent understanding of genomic markers and driver kinases in sarcoma has contributed to the advances in sarcoma therapy with moderate success [9]. Targeted therapies have been employed in many subtypes, particularly in liposarcomas and gastrointestinal stromal tumors (GIST) [10, 11]. CDK4, a cytosolic kinase involved in cell cycle progression, is amplified in greater than 90% of liposarcoma cases. A recent clinical trial of Palbociclib, a selective CDK4 inhibitor, produced a favorable progression-free survival rate in liposarcoma [12]. Similar strategies using small molecule inhibitors and antibody-based therapy against receptor tyrosine kinases (RTKs) have been used with some success in solid tumors [13] including sarcomas [14, 15]. Imatinib mesylate, the first line of therapy for GIST, is a tyrosine kinase inhibitor (TKI) targeted toward c-Kit, c-Abl, and PDGFR, well-characterized oncogenic RTKs. Genotyping GIST allows for directed therapies depending on c-KIT and PDGFR mutational status [10]. The response rates for these targeted therapies are encouraging and pave the way for the development of more specific therapies.

RTKs are key regulators of tumor development as well as metastasis, aiding in the epithelial-mesenchymal transition, migration and angiogenesis. RTK signaling pathways such as VEGFR, PDGFR, and c-Met have been shown to be critical for cell survival, proliferation and metastasis in sarcomas [9, 14]. Current therapies have evolved from single tyrosine kinase inhibitors (TKIs) such as Bevacizumab [16] to angiokinase inhibitors targeting VEGFR in addition to kinases such as PDGFR and c-Kit, such as sunitinib, sorafenib [17] and BIBF1120 [18]. Even though TKIs have been used with moderate success to block tumor cell survival and proliferation, activation of compensatory signaling pathways gives rise to drug resistance in a majority of cases [19, 20]. There is an unmet need for potent multi-targeted kinase inhibitors that may help overcome intrinsic and/or acquired resistance to the traditional targeted therapies.

This present study is the first report describing the *in vitro* and *in vivo* efficacy of MGCD516, a novel, broad spectrum small molecule inhibitor that blocks a wide array of RTKs known to be amplified/overexpressed in sarcomas, including c-Kit, PDGFRβ [21], PDGFRα, c-Met, and Axl [9, 19]. Reports of use of other multi-kinase inhibitors in sarcoma such as imatinib have not been very encouraging [22] or restricted to a smaller patient sub-population such as use of crizotinib in ALK driven tumors [23]. We tested the efficacy of MGCD516 using a wide panel of sarcoma cell lines, including malignant peripheral nerve sheath tumor (MPNST), Ewing sarcoma (A673), osteosarcoma (Saos2), and liposarcoma (DDLS, LS141). Both *in vitro* and *in vivo* efficacy of MGCD516 was significantly better that the other two multi-kinase inhibitors, imatinib and crizotinib. MGCD516 treatment not only inhibits tumor cell proliferation at low nanomolar concentrations *in vitro* but also results significant tumor growth suppression *in vivo* in mouse xenograft models. Our findings strongly suggest further clinical exploration of MGCD516 in patients with soft tissue sarcoma.

## RESULTS

### Basal levels of receptor tyrosine kinase (RTK) expression varies among different sarcoma cell lines

**Figure 1:**
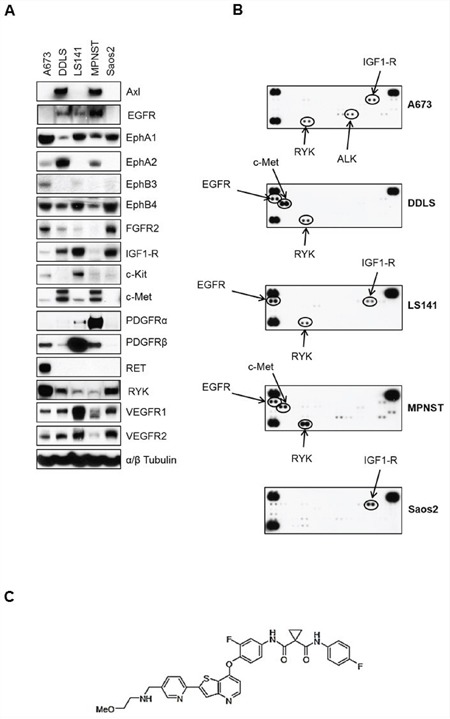
Basal levels of total and phosphorylated receptor tyrosine kinases in five sarcoma cell lines **A.** Approximately, 1 × 10^6^ cells were plated in 60 mm plates in media containing 10%FBS and grown for 24 hours. Next day, 30 micrograms of RIPA lysates were loaded on SDS/PAGE and immunoblotted using indicated antibodies. **B.** 1 × 10^6^ cells were plated in 60 mm plates for 24 hours in media containing 10%FBS. Next day, lysates were prepared according to manufacturer's instructions (R&D Systems, ARY001B) and 200 micrograms of lysates were applied to phospho-RTK membranes overnight at 4°C. Arrows indicate phosphorylated RTK spots in duplicate. **C.** MGCD516 (Sitravatinib) is a novel, potent multi-kinase small molecule inhibitor. Chemical structure of MGCD516 is shown.

Many sarcoma subtypes are characterized by potential driver kinases such as c-Met, IGF1-R, PDGFRα, and c-Kit [9]. To confirm the complexities of sarcoma receptor tyrosine kinase (RTK) signaling, we determined the basal expression levels of RTKs using western immunoblotting in seven different sarcoma cell lines. The RTKs tested included kinases from PDGFR, IGFR, Ephrin (Eph), VEGFR and c-Met family among others. The cell lines tested represent osteosarcoma (Saos2), Ewing sarcoma (A673, CHP100), liposarcoma (DDLS, LS141), malignant peripheral nerve sheath tumor (MPNST), and synovial sarcoma (SYO-1). Based on the results of the preliminary data, we decided to include five cell lines for further studies that showed basal expression levels for the most number of receptor tyrosine kinases (Supplementary Table S1). All five cell lines showed expression of EphA1, EphB4 and VEGFR1 whereas expression levels of kinases from other RTK families varied among different cell lines (Figure 1A). While both liposarcoma cell lines (DDLS and LS141) showed high levels of IGF1-R expression, only MPNST cell line showed significant levels of PDGFRα expression (Figure 1A). We next tested the basal phosphorylation levels of RTKs in these five cell lines grown in 10%FBS containing media using phospho-RTK proteome profiler kit (R&D Systems, ARY001B). Among the cell lines tested, A673, LS141 and Saos2 showed high levels of phospho-IGF1-R, whereas, only two cell lines (DDLS and MPNST) showed high basal phosphorylation levels of c-Met (Figure 1B). Higher auto-radiographic exposures showed presence of basal phosphorylation levels of Eph (Ephrin) receptor kinases as well especially in the cell lines A673 and MPNST (Supplementary Figure S1). Even though we were able to detect basal levels of expression (Figure 1A) and phosphorylation of RYK (Figure 1B), an orphan atypical kinase related to RTKs, it is highly unlikely that it plays any significant role in driving sarcoma cell growth especially since it lacks any catalytic activity of its own [24]. However, it has been shown to be phosphorylated by Eph family of receptors [24]. Taken together, there were multiple potential driver kinases for each cell line with varying degrees of basal expression and phosphorylation levels. The most common RTKs with high basal expression levels in the sarcoma cell lines tested were IGF1-R, c-Kit, c-Met and PDGFRb (Figure 1A). These are also known to be the more canonical sarcoma driver kinases [21, 25, 26].

### MGCD516 is a novel broad-spectrum RTK inhibitor and has potent anti-proliferative effects

**Figure 2:**
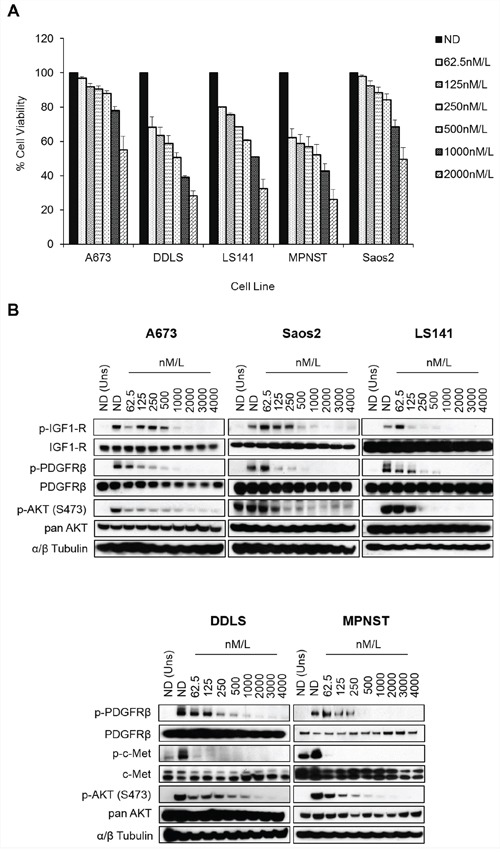
MGCD516 has potent anti-proliferative effect and inhibits multiple RTKs at low nanomolar concentrations **A.** Indicated cell lines were plated in 96-well plates and treated in six wells per condition with increasing doses of MGCD516 for 72 hours. Cell viability was measured using Dojindo Cell Counting Kit 8. Dose response graphs were generated as a percentage of the no drug control. Error bars represent standard error mean. Combined data from three independent experiments is shown. **B.** Indicated cell lines were grown to 60% confluency in serum free media overnight. Next day, cells were treated with indicated concentrations of MGCD516 in serum free media for 3 hours. After the drug treatments, cells were stimulated in drug free media containing 10%FBS. Media containing no FBS was used as unstimulated control. 20 to 30 micrograms of RIPA lysates were loaded on SDS/PAGE and immunoblotted using indicated antibodies. Representative western blots from two independent experiments are shown. ND=No Drug, Uns=Unstimulated control.

Figures 1A and 1B clearly indicate the complexity of signaling pathways in sarcoma cells and demonstrate the need for therapies that target multiple RTKs. MGCD516 is a potent broad-spectrum RTK inhibitor (Figure 1C) with multiple targets including Axl, c-Met, Ephrin receptor family (EphA1, A2, B1, B2, B4), as well as PDGFR and VEGFR family of kinases (Table 1). Many of the targets inhibited by MGCD516 are among the known potential driver kinases in sarcoma [9]. Therefore, we explored anti-proliferative effect of MGCD516 in five sarcoma cell lines used in Figure 1. As shown in Figure 2A, all the cell lines tested were sensitive to increasing concentrations of MGCD516 with three cell lines, DDLS, LS141 and MPNST, showing greater inhibition of proliferation at low nanomolar concentrations than the other two cell lines (A673, Saos2). The IC_50_ values for the three sensitive cell lines (DDLS, MPNST and LS141) ranged between 250–750 nmol/L, whereas, IC_50_ values for the less sensitive cell lines (A673 and Saos2) ranged between 1.5–2.0 mmol/L (Supplementary Table S2). Next we tested the inhibition of RTK signaling by MGCD516 using western immunoblotting by treating the cells with increasing concentrations of the drug. Figure 2B shows complete inhibition of phosphorylation of the targets including c-Met, IGF1-R, and PDGFRb at low nanomolar concentrations ranging from 60–500 nmol/L. In addition to the down-regulation of RTK phosphorylation, we also observed significant down-regulation of p-AKT (Ser473) with increasing concentrations of MGCD516 (Figure 2B).

**Table 1:** *In vitro* kinase inhibition profile of MGCD516 (Sitravatinib)

RTK target	Biochemical (IC_50_ nmol/L)
Axl	1.5
MER	2
MET	20
VEGFR2 (KDR)	5
VEGFR1 (FLT1)	6
VEGFR3 (FLT4)	2
FLT3	8
KIT	6
PDGFRα	30
PDGFRβ	ND
DDR1	29
DDR2	0.5
RET	44
TRKA (NTRK1)	5
TRKB (NTRK2)	9
EPHA2	44
EPHA3	1
EPHA4	76
EPHB2	10
EPHB4	12
FYN	339
RON	43
ROS	59
SRC	156
TIE2	274
YES	298
PYK2	364
BTK	304
EPHB3	249
INSR	5550
HCK	1109
FER	1589
LTK	1938
FES	2010
FMS	2290
ABL	2987
IGF1R	3980
ARG	4098
FGFR1	>5000
FGFR2	>5000
FGFR3	>5000
FGFR4	>5000
ITK	>5000
ERBB1	>5000
ERBB2	>5000
ERBB4	>5000
EPHA1	>10000
EPHB1	>10000
FAK	>10000
JAK1	>10000
JAK2	>10000
JAK3	>10000
ALK	>10000
SYK	>10000
ZAP70	>10000

**Figure 3:**
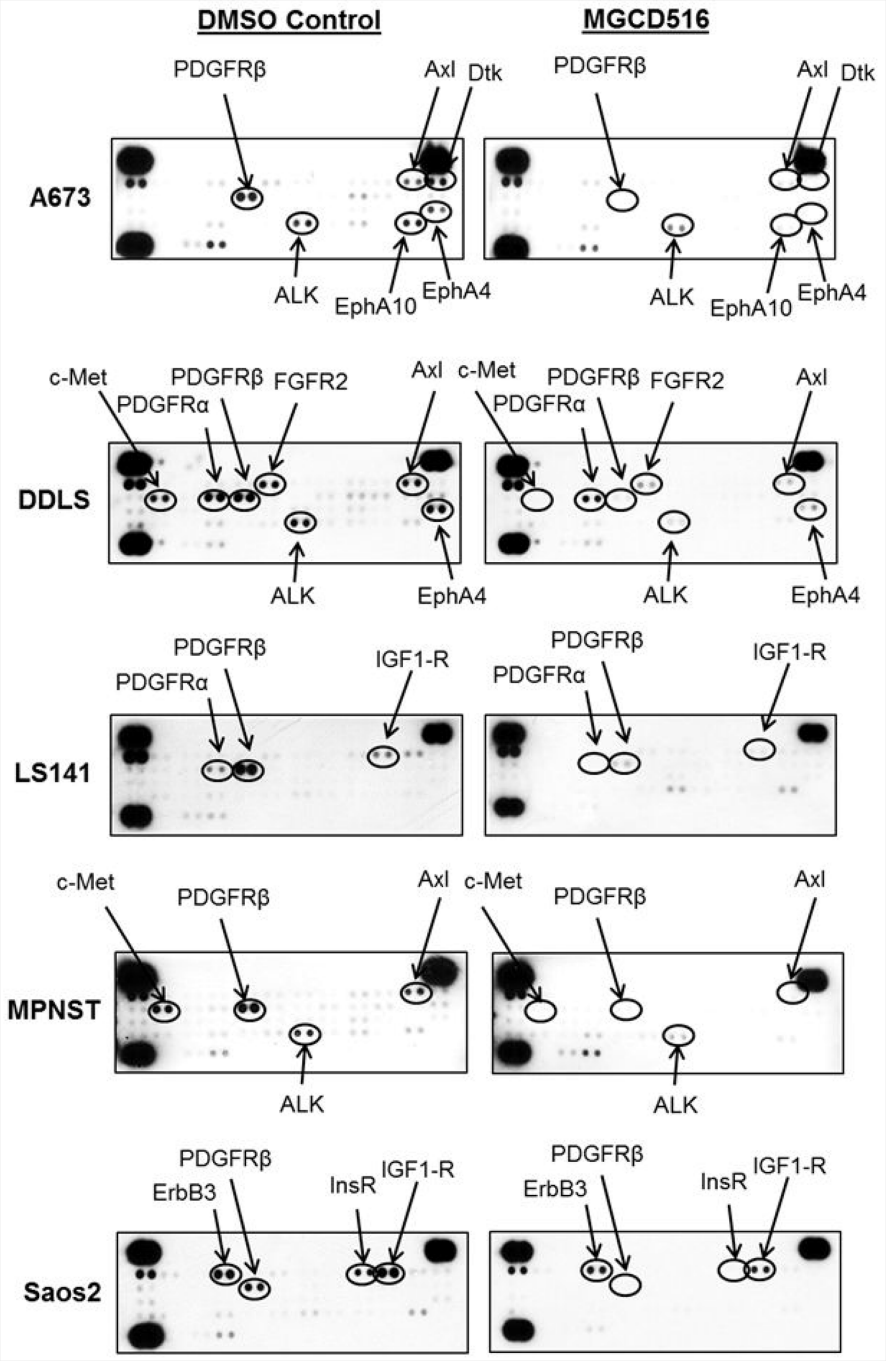
Broad spectrum activity of MGCD516 shows blockade of multiple RTKs across various sarcoma subtypes Approximately, 1 × 10^6^ cells were plated in 60mm plates in serum free media for 24 hours. Next day, DMSO or MGCD516 was added in serum free media and treated for 3 hours. Cells were then stimulated in media containing 10% FBS for 1 hour. Following stimulation, lysates were prepared according to manufacturer's instructions (R&D Systems, ARY001B) and 200 micrograms of lysates were applied to phospho-RTK membranes overnight. Arrows indicate phosphorylated RTK spots in duplicate.

In order to confirm blockade of phosphorylation of multiple RTKs by MGCD516, we next carried out phospho-RTK proteome profiler array analysis. MGCD516 treatment resulted in significant down-regulation of RTK phosphorylation including the canonical RTKs PDGFRα, PDGFRβ, IGF1-R and c-Met (Figure 3). There was also a marked reduction in phosphorylation of RTKs such as Axl and ALK which have also been reported as potential driver kinases in sarcoma [9]. Interestingly, MGCD516 demonstrated inhibition of RTKs including IGF1R and ALK that were not potently inhibited in RTK enzymatic assays (Table 1) suggesting that the inhibitory effects observed for these kinases may be mediated via heterodimerization with direct RTK targets of MGCD516 such as c-Met [27].

Results from cell proliferation, western immunoblotting as well as phospho-RTK array analysis confirmed that MGCD516 is a broad-spectrum multi-kinase inhibitor that results in potent anti-proliferative effects as well as significant down-regulation of its targets at low nanomolar concentrations *in vitro*.

### MGCD516 is superior to other multi-kinase inhibitors in inhibiting cell proliferation, RTK phosphorylation, and phosphorylation of downstream effectors *in vitro*

**Figure 4:**
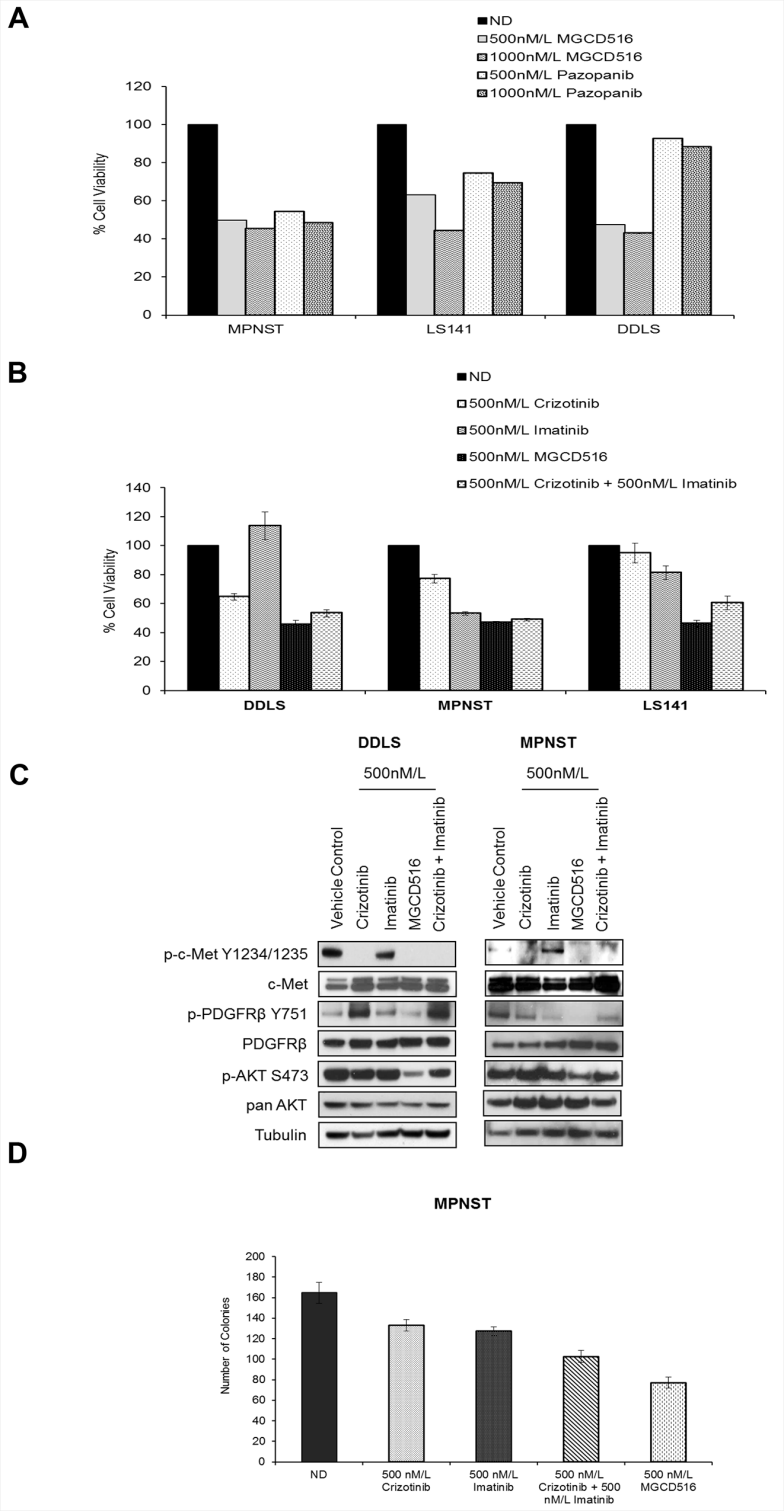
MGCD516 treatment results in superior anti-proliferative effect, better inhibition of downstream targets such as p-AKT and greater reduction in colony growth when compared to imatinib and crizotinib **A.** and **B.** Indicated cell lines were plated in 96-well plates and treated in triplicate with indicated concentrations of drugs for 72 hours. Cell viability was measured using Dojindo Cell Counting Kit 8. Graphs were generated as a percentage of the no drug (ND) control. Error bars represent standard error mean. Combined data from at least two independent experiments is shown. Note: For Figure 4A, error bars are included; however, they are too small to be seen. **C.** Indicated cell lines were grown to 60% confluency in serum free media overnight. Next day, cells were treated with indicated concentrations of MGCD516 in serum free media for 3 hours. After the drug treatments, cells were stimulated in drug free media containing 10%FBS. 30 micrograms of RIPA lysates were loaded on SDS/PAGE and immunoblotted using indicated antibodies. Representative western blots from two independent experiments are shown. **D.** 1,000 cells were plated, in triplicate, onto 100mm dishes and treated the next day with the indicated drugs in media containing 10%FBS for 24 hours. Following treatment, cells were cultured in drug-free media for 10 to 14 days. Colonies were scored with ColCount software from Oxford Optronix (Abingdon, UK). Combined data from the experiment carried out in triplicate is shown.

Next, we compared MGCD516 against other multi-kinase inhibitors, which have overlapping target specificity. First, we evaluated the efficacy of MGCD516 in the three sensitive cell lines (DDLS, LS141 and MPNST) against pazopanib, a drug recently approved for the treatment of sarcoma [28]. Our results (Figure 4A) showed that MGCD516 treatment was able to inhibit proliferation of LS141 and DDLS cell lines significantly better than pazopanib especially at 1000 nM/L concentration (*p*<0.0005) suggesting better pathway inhibition by MGCD516. Inhibition of MPNST cell proliferation was similar for pazopanib and MGCD516 (Figure 4A) given the ability of both pazopanib and MGCD516 to inhibit PDGFR and c-Kit, the two main RTKs driving MPNST cell proliferation [29]. Next, we determined the efficacy of MGCD516 against crizotinib, an ALK and c-Met inhibitor, and imatinib, a PDGFR and c-Kit inhibitor. Based on the IC_50_ value determination (Supplementary Table S2), we decided to use 500 nmol/L of all the tyrosine kinase inhibitors (TKIs). Treatment with MGCD516 showed significant inhibition of proliferation as compared to crizotinib in all the three cell lines tested (*p*<0.005) (Figure 4B). In MPNST, treatment with imatinib showed anti-proliferative effect similar to MGCD516 again highlighting the role of c-Kit and PDGFR as driver kinases in this cell line as has been reported previously [29]. For all three cell lines tested, combined treatment with imatinib and crizotinib showed similar anti-proliferative effect as that of MGCD516 (Figure 4B) strongly indicating that MGCD516 is efficient in blocking multiple RTKs that the other two drugs block individually.

Western blot analysis comparing MGCD516 against imatinib and crizotinib either alone or in combination, in the two sensitive cell lines DDLS and MPNST, showed that MGCD516 was efficient in blocking multiple RTKs and showed significantly better inhibition of downstream effectors such as p-AKT (Ser473) than the other two drugs (Figure 4C). We also compared the efficacy of MGCD516 against imatinib and crizotinib using a colony formation assay as a measure of tumorigenicity. In MPNST cell line, treatment with MGCD516 at 500 nmol/L, concentration similar to the one used for cell proliferation assay comparisons, resulted in significant reduction in colony number compared to no drug control as well as compared to imatinib and crizotinib treatment alone (*p*<0.05, Figure 4D). Similar to the results obtained in Figure 4B, effect of imatinib and crizotinib combination treatment was comparable to MGCD516 alone (Figure 4D). Furthermore, comparison against two structurally similar drugs, carbozantinib [30] and foretinib [31], showed that MGCD516 had a significantly higher anti-proliferative effect (*p* < 0.05, Supplementary Figure S2A).

Many small-molecule RTK inhibitors including sorafenib and lapatinib have been considered as cytostatic agents rather than cytotoxic drugs [32]. To test whether the inhibition of cell proliferation by MGCD516 is due to cytostatic rather than cytotoxic effects, we carried out flow cytometry cell-cycle analysis after MGCD516 treatment in MPNST cell line. Treatment with increasing concentrations of MGCD516 induced a significant G1 cell-cycle arrest (Supplementary Figure S2B, left panel). Down-regulation of cyclin D1 has been reported to play a role in G1 cell-cycle arrest in other cancer cell lines [33]. Western blot analysis after treatment with MGCD516 induced significant down-regulation of cyclin D1 and a significant decrease in hyperphosphorylated form of retinoblastoma protein (p-Rb) but no induction of cleaved PARP was seen (Supplementary Figure S2B, right panel) thus indicating that MGCD516 is a cytostatic than a cytotoxic drug.

Considering the complexities of signaling pathways in sarcoma, we also tested MGCD516 in combination with doxorubicin, a commonly used chemotherapeutic agent. Combination treatment with doxorubicin, at two different concentrations, 10 nM/L and 100 nM/L did not show any significant inhibition of cell proliferation by MGCD516 compared with MGCD516 treatment alone (Supplementary Figure S2C).

### Inhibitory effect of MGCD516 can be replicated by siRNA mediated knockdown of multiple RTKs

**Figure 5:**
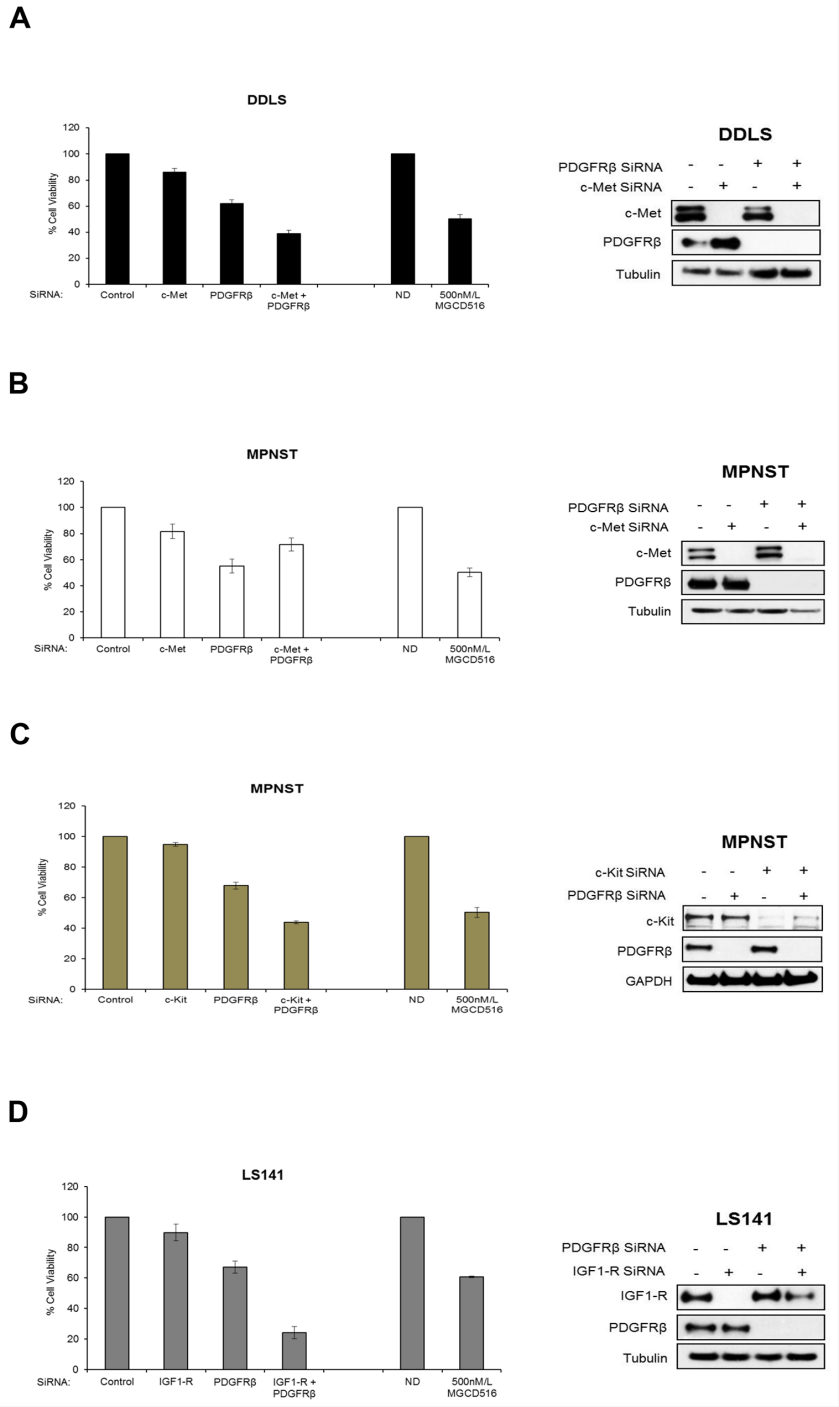
siRNA mediated knockdown of potential driver RTKs results in inhibition of cell proliferation similar to MGCD516 treatment **A, B, C** and **D.** Approximately, 5–7.5 × 10^5^ cells were plated and transfected with 50 nmol/L of indicated siRNAs (GE Dharmacon) the same day. 24 hours later, media was changed and cells were transfected again with 50 nmol/L siRNA. After another 24 hours, cells were harvested and approximately 2000 cells per well were plated in 96 well plates in triplicates. Remaining cells were lysed in RIPA buffer and lysates were used to confirm knockdown of protein expression by western immunoblotting. Cell viability was measured using Dojindo Cell Counting Kit 8. Cell viability assay carried out using MGCD516 treatment at 500 nmol/L is shown for comparison. Error bars represent standard error mean. Combined data from at least two independent experiments is shown.

In order to confirm the role played by potential driver RTKs in each of the three cell lines (DDLS, LS141 and MPNST), we next carried out siRNA mediated knockdown of these kinases either alone or in combination and compared the anti-proliferative effect against MGCD516 treatment. While DDLS and MPNST showed higher basal levels of c-Met and PDGFRβ (Figure 1A), LS141 cell line showed high basal levels of IGF1-R and PDGFRβ. Therefore, we carried out siRNA mediated knockdown of c-Met and PDGFRβ in DDLS and MPNST and IGF1-R and PDGFRβ in LS141 either alone or in combination. Transfection using pooled siRNAs resulted in almost complete knockdown of protein expression (Figure 5A, 5B, 5C and 5D, right panel). Cell viability assays clearly showed that siRNA mediated combined knockdown of the potential driver kinases had a significant anti-proliferative effect compared to scrambled siRNA in all the three cell lines tested (*p*<0.005) (Figure 5A, 5B, 5C and 5D, bar graphs). This anti-proliferative effect was comparable to MGCD516 treatment strongly suggesting that blockade of RTK phosphorylation by MGCD516 is comparable to blockade achieved using siRNA mediated knockdown. For MPNST cell line, combined knockdown of c-Met and PDGFRβ showed lesser anti-proliferative effect (Figure 5B), however, as shown previously [29], combined knockdown of PDGFRβ and c-Kit resulted in similar anti-proliferative effect as that of MGCD516 (Figure 5C) suggesting a role of these two RTKs in driving MPNST cell proliferation. Also, for LS141 cell line, combined blockade of IGF1-R and PDGFRβ, showed significantly higher anti-proliferative effect than 500 nmol/L MGCD516 treatment (Figure 4D). This could in part be due to the fact that the drug concentration used (500 nmol/L) is lower than the IC_50_ concentration observed for LS141 (Supplementary Table S2) as well as higher biochemical IC_50_ value noted for IGF1-R (Table 1) *in vitro*. In addition, as mentioned earlier, IGF1-R may not be a direct target of the drug; instead the effect could be due to blockade of signaling resulting from heterodimerization of IGFR with other RTK targets of MGCD516 [27].

### MGCD516 induces significant tumor growth suppression than imatinib and crizotinib *in vivo*

**Figure 6:**
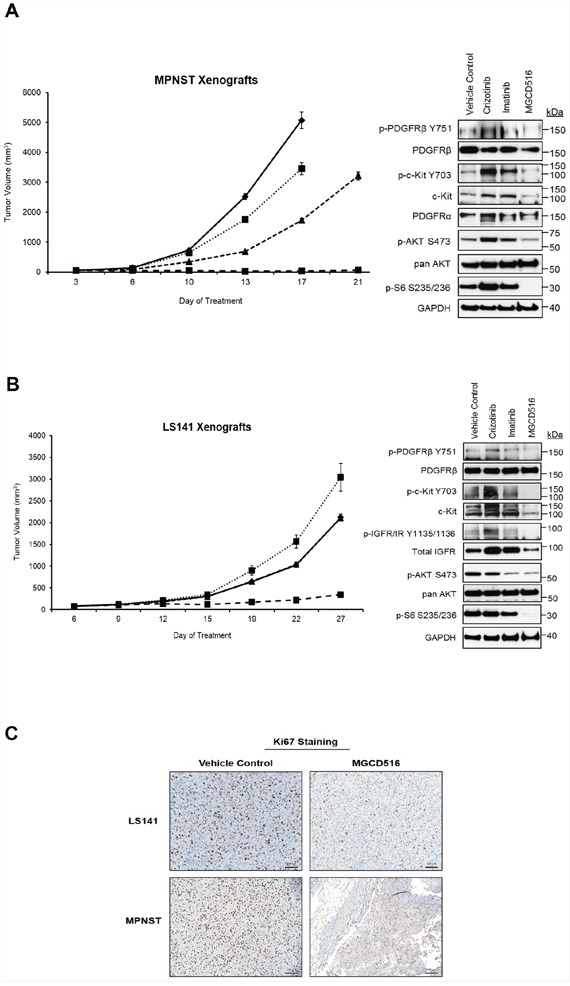
MGCD516 treatment induces significant suppression of tumor growth and better inhibition of downstream targets than imatinib and crizotinib *in vivo* **A, B.** Tumor growth of MPNST and LS141 xenografts treated with the indicated drugs is shown. For MPNST xenografts, treatment was stopped on day 17 for vehicle control and crizotinib treatment group (as the tumors were huge). Note: In the graph for LS141 xenografts, the tumor growth volume lines for vehicle control and imatinib treatment groups are overlapping and therefore not visible as two separate lines. 30 micrograms of RIPA lysates obtained using sample grinding kit (GE Healthcare) from xenograft tissues at the end of 3 week treatment were loaded on SDS/PAGE and immunoblotted using indicated antibodies. **C.** Xenograft tissues obtained from mice at the end of 3 week treatment with vehicle or MGCD516 were stained immunohistochemically using Ki67 antibody. Scale bar (100 μm) is shown in the lower right hand corner of each image. Representative image from at least 2 animals sacrificed at the end of drug treatment is shown.

Next, we carried out *in vivo* mouse xenograft experiments using MPNST and LS141 models. In order to be consistent with our *in vitro* data, we compared MGCD516 treatment against imatinib and crizotinib *in vivo*. Treatment with any of the drugs including MGCD516 did not result in toxicity as determined by animal weight during the study period (Supplementary Figure S3). In both MPNST and LS141 xenograft experiments, MGCD516 treatment resulted in significant suppression of tumor growth compared to vehicle control (*p*<0.0005) as well as compared against imatinib and crizotinib (Figure 6A and 6B) (*p*<0.005). Western blot analysis of tissue samples obtained after 3 weeks of treatment showed greater inhibition of phosphorylation of potential driver kinases such as c-Kit, IGF1-R and PDGFRβ compared to imatinib and crizotinib as well as significant down-regulation of downstream effector pathways such as p-AKT (Ser473) and p-S6 (Ser235/236) (Figure 6A and 6B). To test the effect of MGCD516 treatment on proliferating tumor cells, we tested Ki67 as a proliferation marker on formalin fixed tumor tissues. Tumor samples treated with MGCD516 for 3 weeks showed significantly less Ki67 staining compared to vehicle control (Figure 6C). Reduction in Ki67 staining indicated strong anti-proliferative effect of MGCD516.

## DISCUSSION

Sarcomas represent a diverse set of malignancies that are difficult to treat given the complexities of various subtypes. Sarcomas are traditionally categorized into two broad categories: tumors with translocations or activating mutations, whereas, the second category includes more complex tumors showing multiple genomic aberrations [34]. Genetically more complex group includes some of the more commonly diagnosed sarcomas such as leiomyosarcoma, and osteosarcoma. Many sarcoma subtypes belonging to both these categories show aberration and/or mutation in signal transduction pathways, particularly defects in receptor tyrosine kinase (RTK) signaling [34, 35]. Recent evidence suggests involvement of RTKs such as PDGFR [29, 36, 37], c-Met [38], Axl [39], IGF1-R [40], EphB4 [41] and ErbB4 [42] as driver kinases in different sarcoma subtypes including synovial sarcoma, Ewing sarcoma as well as malignant peripheral nerve sheath tumor (MPNST). Detailed phosphoproteomic analysis from cell lines and patient tumors also revealed involvement of RTKs such as PDGFR, Eph receptors, Axl, c-Met and IGF1-R [9] as potential driver kinases in sarcomas. Even though RTKs present an attractive therapeutic target [14], in many cases, cells rapidly acquire resistance via compensatory activation and/or mutation of signaling pathways [43]. Therefore, there is an urgent need to develop novel therapeutic agents that will target multiple RTKs in order to block such compensatory signaling pathways thereby preventing development of drug resistance.

MGCD516 is a novel, small molecule inhibitor that has broad spectrum activity against many of the canonical RTKs including c-Met, VEGFR, Axl, Ephrin receptors (Eph), c-Kit and PDGFR. The biochemical IC_50_ values for many of these RTKs lie in the low nanomolar range (1–200 nmol/L). In the present study, we were able to detect varying levels of expression and phosphorylation of many of the MGCD516 targets such as c-Met, Axl and PDGFR as well as VEGFR and Eph receptors in all five sarcoma cell lines tested. When we evaluated the efficacy of MGCD516 in a cell proliferation assay, we observed increased sensitivity with increasing doses of the drug with IC_50_ values ranging in the low nanomolar range (250–800 nmol/L) for three out of five cell lines (DDLS, LS141 and MPNST). It must be noted though that two of the five cell lines tested (A673 and Saos2) were less sensitive to inhibition by MGCD516 than the other cell lines. This could probably be due to the fact that A673 cell line, in addition to having high basal expression and phosphorylation levels of many RTKs (IGF1-R, ErbB4, EphA4, EphA10, and ALK), also carries a (BRAF *V600E*) mutation which could potentially play a role in reduced sensitivity to the drug. For Saos2 cell line, not only were we able to detect lesser number of MGCD516 targets on western blot but also high basal phosphorylation levels of IGF1-R on phospho-RTK array. *in vitro* IC_50_ value for IGF1-R inhibition by MGCD516 is much higher (3980 nmol/L) compared to other RTKs such as c-Met (20 nmol/L) or PDGFRα (30 nmol/L) indicating that cell lines with IGF1-R as the sole driver kinase may exhibit reduced sensitivity to the drug. Direct inhibition of IGF1-R activity was not seen in the *in vitro* enzymatic assays, suggesting that the reduction in phosphorylation could be a result of blockade of heterodimerization with other MGCD516 targets such as c-Met [27].

Multi-kinase inhibitors including imatinib, which blocks RTKs such as c-Kit and PDGFR, have been used in sarcoma clinical trials with modest success [22]. Crizotinib, which inhibits c-Met and ALK, is also being tested in clinical trials (Clinical Trial Identifier NCT01524926) for sarcoma. Pazopanib, an anti-angiogenic agent, that blocks multiple driver RTKs including c-Kit, VEGFR, PDGFR as well as kinases from FGFR family, was recently approved for the treatment of soft-tissue sarcoma [28, 44]. However, one of the reasons for limited success with such multi-kinase inhibitors is the ability of tumors to activate alternate/escape pathways that are not the primary targets of the drug. MGCD516 is unique in a way that it has broad spectrum activity against many RTKs including c-Met, c-Kit, Axl, PDGFR, and Eph receptors that are known to play a role in driving sarcoma cell growth, and thus, it is able to inhibit many of the internal compensatory pathways. Our studies comparing MGCD516 against imatinib and crizotinib showed that MGCD516 was clearly superior to the other two drugs in terms of inhibition of cell proliferation as well as blockade of signaling pathways. Interestingly, combination of imatinib and crizotinib showed similar anti-proliferative effect as that of MGCD516 alone strongly suggesting that MGCD516 is able to block multiple RTKs that imatinib and crizotinib block individually.

In the present study, we also carried out silencing of the target RTKs using siRNA mediated approach to verify that blockade of RTK signaling by MGCD516 was indeed responsible for inhibition of cell proliferation in DDLS, LS141, and MPNST cell lines. Combination of siRNAs against potential driver RTKs resulted in comparable inhibition of cell proliferation as that of MGCD516 treatment. In MPNST cell line, combined knockdown of PDGFRβ and c-Kit but not c-Met resulted in similar anti-proliferative effect as that of MGCD516 indicating a role of these two RTKs in driving MPNST cell proliferation as shown previously [29]. In LS141 cell line, siRNA mediated knockdown of IGF1-R and PDGFRβ resulted in higher inhibition of proliferation than MGCD516 treatment, presumably due to the fact that the IC_50_ value for IGF1-R inhibition by MGCD516 is much higher than for other kinases. Nevertheless, these results clearly indicate that MGCD516 is efficient in blocking multiple pathways that are critical for sarcoma cell proliferation and this effect can be mimicked by siRNA mediated knockdown of individual RTKs.

Similar to the anti-proliferative effect observed, MGCD516 showed significant inhibition of colony growth and has a greater inhibitory effect on cell colonization than imatinib and crizotinib treatment alone, potentially owing to its greater broad-spectrum activity.

Sustained inhibition of RTKs such as c-Kit and PDGFR has been shown to suppress tumor growth in MPNST [29]. Results from our *in vivo* data showed that MGCD516 treatment results in significant suppression of tumor growth compared to the other two drugs in both MPNST as well as LS141 xenograft models. Immunohistochemical studies using Ki67 staining as a marker for cell proliferation showed that MGCD516 treatment indeed resulted in decreased cellular proliferation. Western immunoblotting showed that MGCD516 treatment not only resulted in significant blockade of phosphorylation of multiple RTKs such as PDGFR, c-Kit and IGF1-R but also inhibited downstream effectors such as p-AKT and p-S6 which are critical for tumor cell survival and proliferation. Since MGCD516 can inhibit RTKs such as PDGFR and VEGFR, we speculate that the significant tumor suppression achieved *in vivo* is not only limited to inhibition of signaling pathways in tumor cells but also due to an effect on stromal cell signaling mediated via these kinases. Even though MGCD516 does show inhibition of VEGFR family *in vitro*, differentiated effects are observed from other more well-studied VEGFR inhibitors such as sunitinib, which do not share additional MGCD516 targets (personal communication with Dr. James Christensen, Mirati Therapeutics). Interestingly, crizotinib treatment in LS141 xenografts showed greater tumor growth than vehicle control perhaps as a result of activation of other RTKs such as increased phospho-c-Kit and phospho-IGF1-R signal seen on western immunoblotting.

Taken together, our data strongly suggest that broad spectrum inhibition of multiple RTK signaling pathways by MGCD516 both *in vitro* and *in vivo* results in superior activity compared to imatinib and crizotinib. Significant down-regulation of downstream pathways such as p-AKT and p-S6 also contributed to higher tumor growth suppression *in vivo*. This is the first pre-clinical report describing MGCD516 (Sitravatinib) as a potent and broad spectrum inhibitor of RTKs. A phase I trial with MGCD516 is already underway and results from this present study can be extended to other RTK driven solid tumors. We believe that our data would support the development of MGCD516 as a potential therapy for patients with soft-tissue sarcoma.

## MATERIALS AND METHODS

### Drugs

MGCD516 (Sitravatinib) was provided by Mirati Therapeutics (San Diego, CA) in the form of powder; it was dissolved in dimethyl sulfoxide (DMSO) for *in vitro* studies. Vehicle and MGCD516 were prepared to a final concentration of 0.5% hydroxypropyl methylcellulose (HPMC) and 0.1% Tween-80 solution (pH 1.4) for xenograft studies. Both imatinib and crizotinib, purchased from LC Laboratories (Woburn, MA), were dissolved in DMSO for *in vitro* experiments and formulated in water for *in vivo* experiments.

### Cell lines

Malignant peripheral nerve sheath tumor cell lines (MPNST, ST8814) were supplied by Dr. Jonathan Fletcher (Dana Farber Cancer Institute, Boston, MA). MPNST and ST8814 cell lines were authenticated as previously described [29]. Ewing sarcoma (CHP100, A673) cell lines were obtained from Dr. Melinda S. Merchant (Center for Cancer Research, NCI/NIH, Bethesda, MD). Dedifferentiated liposarcoma cell lines (LS141, DDLS) were obtained from Dr. Samuel Singer [Memorial Sloan Kettering Cancer Center (MSKCC), New York, NY], and were authenticated by gene expression profiling before distribution [45]. Synovial sarcoma cell lines (SYO-1 and HSSY-II) were obtained from Dr. Marc Ladanyi (MSKCC). Osteosarcoma cell line (Saos2) was obtained from ATCC. All cell lines except SYO-1 were cultured in RPMI media with 10% FBS, 100 U/mL penicillin, and 100 mg/mL streptomycin, maintained at 37°C in 5% CO2, and passaged for no more than 4 months. SYO-1 cell line was maintained in DME media with 10% FBS, 100 U/mL penicillin, and 100 mg/mL streptomycin, maintained at 37°C in 5% CO2. Initial stocks of all cell lines were received from their sources within the past 3 years. Cell lines CHP100 and A673 were authenticated using RT-PCR, and found to have their expected characteristic chromosomal translocations. SYO-1 and HSSY-II cell lines were authenticated by confirming the expression of the pathognomonic SYT-SSX fusion gene by RT-PCR. All cell lines were determined to be mycoplasma free via testing in the MSKCC Monoclonal Antibody Core Facility using biochemical assay MycoAlert.

### Cell viability assay

Cell viability assays were carried out with the Dojindo Molecular Technologies Kit per manufacturer's instructions. Briefly, 2,000–3,000 cells were plated in 96-well plates in RPMI/DME media with 10% FBS and then treated with the indicated drugs the next day. After 72 hours, media was replaced with 100 μL of media with 10% serum and 10% CCK-8 solution (Dojindo Molecular Technologies Kit). After 1 hour, the optical density was read at 450 nm using a Spectra Max 340 PC (Molecular Devices Corp.) to determine viability. Background values from negative control wells without cells were subtracted for final sample quantification. Data was plotted as % cell viability compared to DMSO (No Drug, ND) control. IC50 was extrapolated from cell viability data using CompuSyn software according to the manufacturer's instructions.

### Western blots

Western blots were carried out as previously described [46]. Briefly, cell lysates were prepared by washing the cells once with sterile PBS followed by scraping in radioimmunoprecipitation assay (RIPA) lysis buffer. For xenograft tissues, lysates were prepared by cutting the snap frozen tumor tissue into a small piece and then grinding it in RIPA lysis buffer using sample grinding kit (GE Healthcare). Protein concentrations were measured using Bio-Rad protein assay dye (Bio-Rad) and equal amounts of protein (20–30 mg) were loaded on 4%-12% gradient gels (Invitrogen) and transferred to PVDF membrane (Immobilon, Millipore) or Nitrocellulose (ThermoFisher) for the detection of phosphorylated c-Kit. After blocking with 5% milk, membranes were probed with primary antibodies overnight at 4°C. Bound antibodies were detected with horseradish peroxidase secondary antibodies (GE Healthcare) and visualized by enhanced chemiluminescence reagent (GE Healthcare). Antibodies for western immunoblotting were purchased from Cell Signaling Technology unless otherwise mentioned.

### Human phospho-RTK array

Phospho-RTK analysis was carried out according to manufacturer's instructions (R&D systems, ARY001B). Briefly, 1 × 10^6^ cells were plated in no serum media overnight (unless otherwise indicated) in 60mm plates. Next day, indicated concentrations of drugs were added in no serum media. After 3 hours of treatment, cells were stimulated using 10%FBS containing media (no serum media was used as unstimulated control) for 1 hour. Cells were then washed once with sterile PBS, lysed in NP40 lysis buffer and approximately 200–300 μg of lysates were loaded on blocked phospho-RTK array membranes overnight. Phospho-RTK array co-ordinates can be found at www.rndsystems.com/pdf/ary001B.pdf.

### Gene silencing

50 nM siRNAs (pooled siRNAs, Dharmacon) specific for human IGF-1R, c-Met and PDGFRβ were transfected using Lipofectamine RNAiMAX reagent (Invitrogen). Scrambled (non-targeting pool) siRNA was used as control. 48 hours after transfections, cells were trypsinized, counted using a Nexcelom cell counter and plated in 96-well plates for cell viability assays as described. Cells were also collected, lysed and analyzed by western blot to check for knockdown of protein expression.

### Colony assay

1,000 cells were plated, in triplicate, onto 100 mm dishes and allowed to adhere overnight. Cells were treated for 24 hours with the indicated drugs. Following treatment, cells were cultured in drug-free media for 10 to 14 days. The resulting colonies were fixed in 20% ethanol and stained with 2.5% crystal violet. Colonies were scored with ColCount software from Oxford Optronix (Abingdon, UK).

### Xenograft studies

Briefly, LS141 and MPNST xenografts were transplanted subcutaneously in the flank of ICR/SCID mice. Once tumors reached a volume of 80–100 mm^3^, the mice were randomized into different groups of 7–10 animals each and treated with the indicated drugs or vehicle control. Crizotinib was dosed at a concentration of 50 mg/kg i.p. QD, 5 days a week. Imatinib was dosed at a concentration of 30 mg/kg i.p. QD, 5 days a week. MGCD516 was dosed at a concentration of 15 mg/kg p.o. QD, 5 days a week. Tumor size was measured twice weekly by caliper. The average tumor volume in each group was expressed in cubic millimeter and calculated using the formula p/6 × (large diameter) × (small diameter)^2^. After 3 weeks of drug treatment, 3 animals in each group were sacrificed and the resected tumors were divided for formalin fixation (for hematoxylin/eosin staining and IHC) and snap frozen tissue (for western blot). Experiments were carried out under an Institutional Animal Care (MSKCC) and Use Committee–approved protocol, and institutional guidelines for the proper and humane use of animals were followed.

### Immunohistochemistry

Preparation of tissue sections for IHC and staining for Ki67 was carried out by MSKCC molecular cytology core facility and has been described previously [29].

### Statistical analysis

*in vitro* experiments were carried out at least 3 times unless otherwise indicated. Error bars shown in the graphs represent standard error. Graphs were plotted using Microsoft Excel. Statistical analyses were carried out using student's *t*-test with 95% confidence interval.

## SUPPLEMENTARY FIGURES AND TABLES


