# Supplementary material for: Significant blockade of multiple receptor tyrosine kinases by MGCD516 (Sitravatinib), a novel small molecule inhibitor, shows potent anti-tumor activity in preclinical models of sarcoma
Source: Oncotarget. 2015 Dec 10;7(4):4093–109. doi: 10.18632/oncotarget.6547 (PMC4826192; doi:10.18632/oncotarget.6547)
Supplement: Supplementary file 1 [file oncotarget-07-4093-s001.pdf]

SUPPLEMENTARY FIGURES AND TABLES

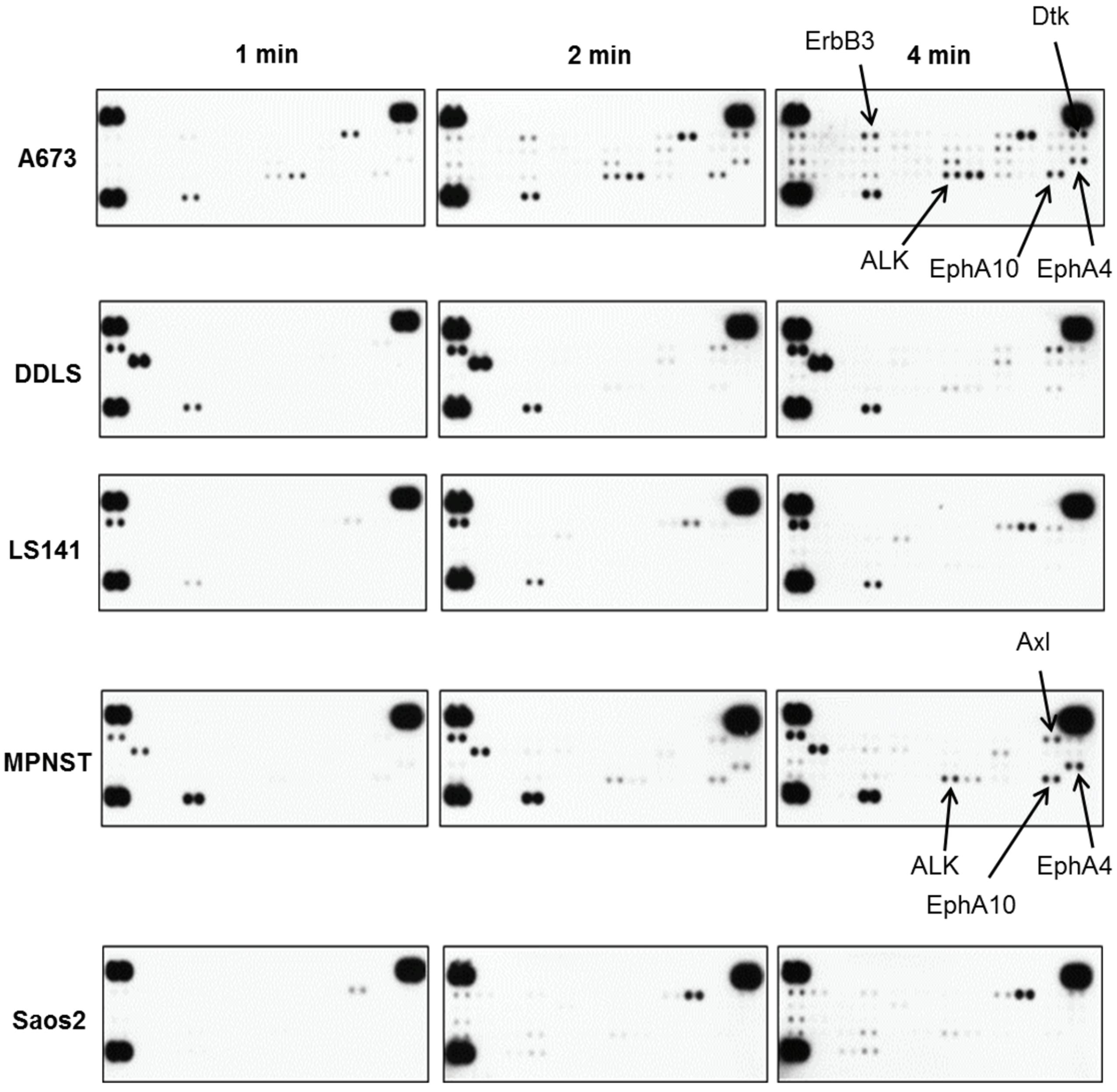

Supplementary Figure S1: Basal levels of phosphorylated receptor tyrosine kinases in five sarcoma cell lines. Additional autoradiographic exposures for experiment shown in Figure 1B. Phosphorylated RTKs are labeled with an arrow.

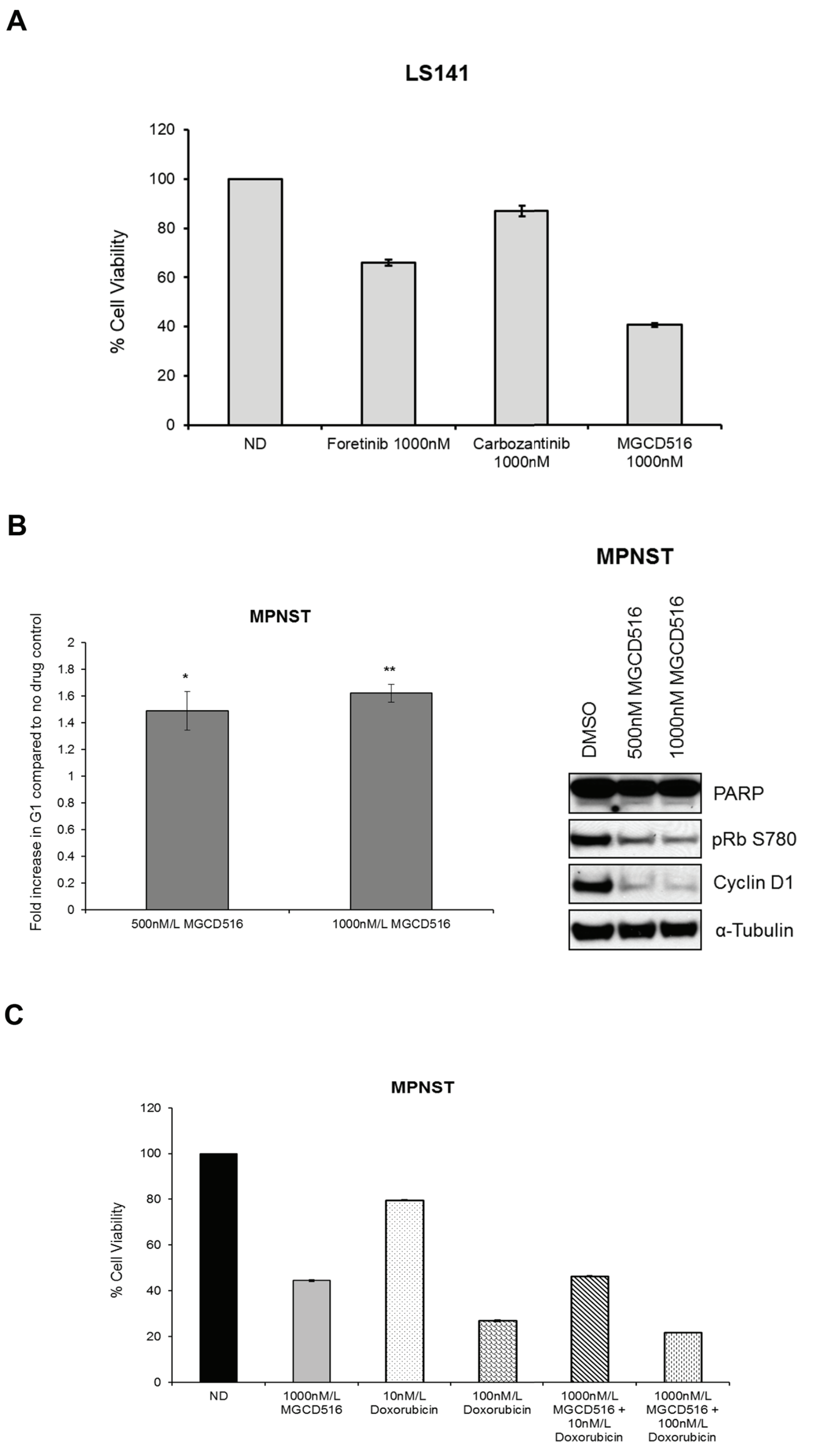

Supplementary Figure S2: A. LS141 cells were plated in 96-well plates and treated in triplicate with indicated concentration of drugs for 72 hours. Cell viability was measured using Dojindo Cell Counting Kit 8. Error bars represent standard error mean. Combined data from at least two independent experiments is shown. **B.** The cell cycle effects of MGCD516 treatment is shown in MPNST cells as fold increase in G1 phase compared to no drug control. * = $p<0.05$, ** = $p<0.005$. Error bars represent standard error mean. Combined data from two independent experiments is shown. Western blot analysis of cell lysates shows that pRb and cyclin D1 are down-regulated in cells treated with MGCD516. **C**. MPNST cells were plated in 96-well plates and treated in triplicate with indicated concentrations of drugs for 72 hours. Cell viability was measured using Dojindo Cell Counting Kit 8. Error bars represent standard error mean. Combined data from two independent experiments is shown.

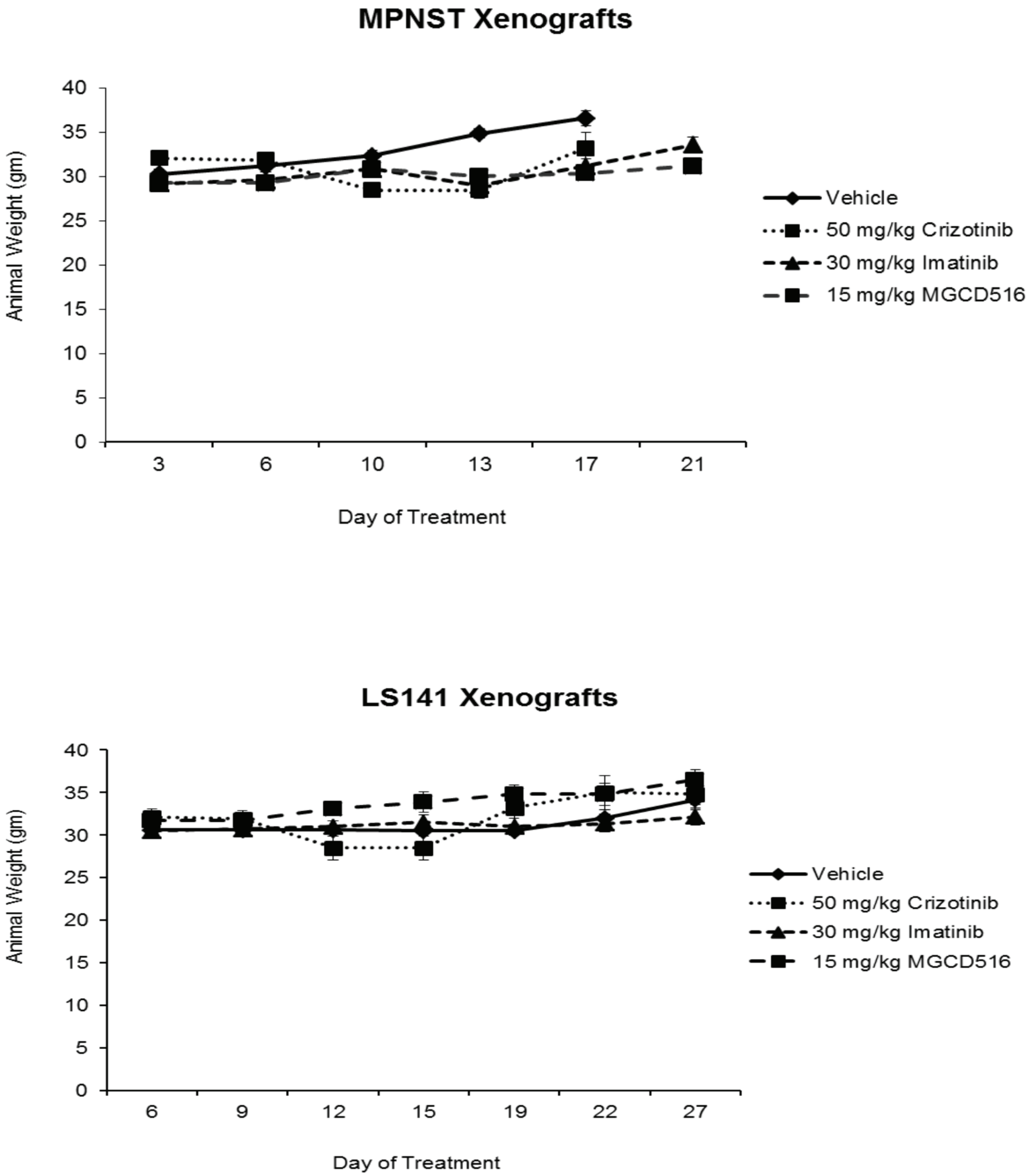

Supplementary Figure S3: Animal weight (gm) monitored as a measure of toxicity throughout the drug treatment is shown.

Supplementary Table S1: Number of kinases detected on western blot for each cell line

Cell line	Number of kinases detected on western blot (Basal expression)
A673	13
LS141	13
MPNST	13
DDLS	12
Saos2	8

Supplementary Table S2: IC50 values for cell proliferation

Cell line	Approximate IC_{50} (nmol/L)
A673	1750
DDLS	340.1
LS141	705.7
MPNST	266
Saos2	1830
